# Commentary: Sex Differences in the Peripheral Immune System in Patients with Depression

**DOI:** 10.3389/fpsyt.2017.00145

**Published:** 2017-08-09

**Authors:** Jonas Breidenstein, Charlotte Przyborowski, Andreas Walther

**Affiliations:** ^1^Biological Psychology, Technische Universität Dresden, Dresden, Germany

**Keywords:** depression, major depressive disorder, sex differences, inflammation, stress, cortisol, hypothalamic–pituitary–adrenal axis, glucocorticoid receptor

## Introduction

Statistics report a three to four times increased likelihood of developing a depressive episode in individuals afflicted with systemic inflammation (1), showing higher levels of inflammatory markers such as C-reactive protein (CRP) or interleukin-6 (IL-6) in patients with major depressive disorder (MDD) (2). However, the molecular mechanisms underlying this relationship remain to be further investigated (3, 4). Women show much higher rates of major depression than men (5, 6), although again, the underlying mechanisms are a matter of current debate (7–9). In addition, women show an increased prevalence of autoimmune disease compared to men (10). Therefore, it seems plausible to reason that sex differences may play a role in the relationship between MDD and inflammation. Previous studies investigating differences in inflammatory markers between women and men with MDD have yielded inconsistent results (11–13). There is also mounting evidence linking inflammatory markers to suicidal behavior (14–16). Sex differences in suicidal behavior are reported, with women showing more suicide attempts, while rates of completed suicide are much higher in men (17–19). Although lassitude and pessimism are prominent in depressed patients with suicidal ideation, it is seldom reported whether inflammation contributes to these two features of depression (20). Moreover, different levels of circulating inflammatory markers such as CRP and IL-6 are reported in men and women, further underlining the necessity to investigate gender differences in this field (21, 22). The relationship between sex differences and inflammation levels in patients with MDD is, therefore, of high interest, as identifying potential biomarkers leading to the development and progression of depression would not only improve our understanding of the disease but might also open up promising avenues for prevention and treatment.

Therefore, in their recently published study, Birur and colleagues investigated gender differences with regard to the link between MDD and inflammation (23). The authors hypothesized that high levels of inflammatory markers in depressed women compared to depressed men would be present, as well as higher levels of inflammatory markers in depressed women compared to female control participants.

## Measures and Approaches

The study sample comprised 200 participants aged between 19 and 65 years and was divided into four groups: depressed males, male controls, depressed females, and female controls. Inclusion criteria such as good physical health and stable medical condition, and a wide range of exclusion criteria (intake of corticosteroids, antibiotics, or anti-inflammatory medication, current infectious diseases, history of autoimmune or inflammatory disorders) prevented confounding of the inflammatory markers in the serum of the participants. Included (anti-/pro-) inflammatory markers were interferon-γ (IFN-γ), IL-1β, IL-2, IL-5, IL-6, IL-8, IL-10, IL-12, p70, IL-17, tumor necrosis factor alpha (TNF-α), adiponectin, and leptin. The severity of individual depressive symptoms was assessed by the Montgomery–Åsberg Depression Rating Scale (MADRS), including the following dimensions: apparent sadness, reported sadness, inner tension, reduced sleep/appetite, concentration difficulties, lassitude, inability to feel, pessimistic thoughts, and suicidal thoughts. After controlling for age and body mass index (BMI), the variables of interest were analyzed using an ACONVA to compare depressive males and females to their corresponding controls.

## Effects of Sex on Inflammatory Makers and Symptoms of Depression

As hypothesized by Birur and colleagues, no significant differences between depressed males and control males were identified with regard to any of the inflammatory markers (23). In contrast, depressed women showed higher serum levels of the pro-inflammatory markers IL-8, IFN-γ, and leptin compared to healthy control women. Moreover, adiponectin, a known anti-inflammatory marker (24), was significantly reduced in depressed females compared to healthy control females. Although this finding supports the authors’ hypothesis that depressed women show elevated inflammatory levels compared to healthy control women, it should be noted that these are just four out of 13 serum markers. On the other hand, somewhat surprisingly, lower levels of the pro-inflammatory cytokine IL-5 were reported for depressed females when compared to healthy control females. The direct comparison of depressed males and depressed females only revealed higher levels of IL-6 and leptin in women after adjusting for BMI. The gender-specific comparison of depressive symptoms revealed that depressed females score significantly higher on the lassitude, pessimism, and suicidal thoughts dimensions of the MADRS. Depressive symptoms were only significantly related to IL-12 in men, while in women, significant correlations were identified between depressive symptoms and IL-1 and TNF-α.

## Exploring Hypotheses and Conclusion

Taking everything into consideration, the study by Birur and colleagues has provided valuable new insights concerning sex differences in the pathophysiology of depression (23). These insights do indeed indicate that women show higher levels of inflammation during depression. However, it is not possible to draw conclusions about causal relations from these results, as the data were of a cross-sectional nature. Therefore, the reasoning that elevated inflammation might contribute to depression should be considered with caution despite findings reporting higher susceptibility to depressive symptoms in women with elevated CRP levels (25). Further research is needed to investigate this question from a longitudinal perspective. Surprisingly, the authors did not identify elevated levels of IL-6 in depressed women compared to control women nor a positive correlation between IL-6 and depression severity, although there is a great body of literature showing IL-6 to be elevated in depression (3). This inconsistency might be due to a potential bias in the sample selection including in- and outpatients as well as community dwellers, or having generally older participants in the MDD groups compared to the control group (although it has been controlled for age in the analyses). Studies have reported age-related alterations in steroid levels or body composition to be intensified by depressive symptoms (26, 27). Furthermore, not taking different mood states of the subjects into account could be an additional reason for the reported finding.

Importantly, when investigating sex differences in inflammation-related disorders, we need to acknowledge the anti-inflammatory effects of androgens and the inflammation-enhancing effects of estrogens (28). These might underlie and potentially explain the identified sex differences in inflammation and MDD. Furthermore, stress has not been accounted for as a possible influencing factor or mediator in the relationship between inflammation and depression (29). A variety of studies have shown that stress has important effects on the level of several inflammatory markers (30, 31). Importantly, one of the most consistent findings in depression research is hyperactivity of the hypothalamic–pituitary–adrenal (HPA) axis with resulting elevated levels of cortisol—the end product of the HPA axis (32). Although HPA axis activation has immunosuppressive effects, in MDD, both elevated cortisol levels and elevated inflammation are present due to the loss of the anti-inflammatory properties of the glucocorticoid receptor (GR) (3). Capturing this time-dependent change from anti-inflammatory to potentially pro-inflammatory properties of the GR in MDD represents one of the most important goals for research on the pathophysiology of depression, and might substantially advance the clinical use of biomarkers for MDD. A strong interconnectedness has been consistently shown between chronic psychosocial stress, which is strongly related to MDD, and elevated inflammation (33). Most recently, Mondelli and colleagues reported psychosocial stress to be one of the main factors determining neuroinflammation (4). Thus, although the study by Birur and colleagues has shed new light on the question of sex differences in depression, future studies should focus on the longitudinal co-regulation of inflammatory and glucocorticoid levels in order to investigate the question of causality. Moreover, these studies should also include psychosocial stress measures, as well as measures for HPA axis activity and function.

## Author Contributions

JB wrote the first draft of the manuscript. CP critically revised the first version of the manuscript. AW critically revised the manuscript and edited it to its final version.

## Conflict of Interest Statement

The authors declare that the research was conducted in the absence of any commercial or financial relationships that could be construed as a potential conflict of interest.
